# Retrospective comparison of conservative treatment and surgery for widely displaced olecranon fractures in low-demanding geriatric patients

**DOI:** 10.1007/s00402-021-04031-7

**Published:** 2021-07-05

**Authors:** Peter Kaiser, Kerstin Stock, Stefan Benedikt, Tobias Kastenberger, Gernot Schmidle, Rohit Arora

**Affiliations:** Department of Orthopaedics and Traumatology, Medical University of Innsbruck, Anichstr. 35, 6020 Innsbruck, Austria

**Keywords:** Olecranon, Fracture, Elderly, Geriatric, Mayo, Treatment, Surgery, Conservative

## Abstract

**Introduction:**

The aim of this study was to evaluate the difference of the clinical outcome of elderly patients who were treated surgically or conservatively for a displaced olecranon fracture (Mayo type IIA or IIB).

**Patients and methods:**

Patients above the age of 70 years who were treated surgically (*n* = 11) for a displaced Mayo type IIA and IIB olecranon fracture between July 2015 and February 2019 were retrospectively compared with patients who were treated conservatively (*n* = 6). The range of motion, elbow strength, grip strength, VAS, DASH, OES, MEPI and Broberg and Morrey scores were evaluated.

**Results:**

The conservative group showed a non-union with a persistent fracture gap of 17 mm (SD 12 mm) at the articular rim and 31 mm (15 mm) at the dorsal rim while there was no case of non-union in the surgical group. The arch of motion was 120° in the conservative group and 136° in the surgical group. There was no obvious difference in elbow extension strength in comparison to the healthy contralateral side (*p* = 0.20; 88% group I/87% group II). There was no difference in the OES (*p* = 0.30; 42 (SD 7) vs. 45 (SD 5)) and MEPI score (*p* = 0.46; (SD 8) vs. 96 (SD 19)). The conservative group presented a slightly worse DASH [*p* = 0.10; 26 (SD 25) vs 7 (SD 14)] and a significantly worse Broberg and Morrey score (*p* = 0.02; 84(SD 9) vs. 95 (SD 7)). The conservative group presented one complication (ulnar nerve palsy), while the surgical group presented two cases (prolonged lymphedema; blocked forearm rotation due to screw length with consecutive revision surgery).

**Conclusion:**

Widely displaced olecranon fractures can successfully be treated conservatively in low-demanding geriatric patients with a satisfactory outcome. Patient selection is essential as patients that are more active might benefit from surgical treatment. Yet, treatment risks and benefits need to be balanced carefully in regard to the patient`s demands and requests.

## Introduction

Olecranon fractures account for app. 18% of all proximal forearm fractures with an incidence of 12 per 100,000 persons. There is a steep increase of the incidence at the age above 60 years for women and above 80 years for men up to an incidence of 65–80 per 100,000 persons [1].

The most common cause of fracture is a fall from standing height with a direct impact to the olecranon [1, 2]. A powerful contraction of the triceps muscle during a fall on the outstretched arm has also been described as a common trauma mechanism [2–4]. Displacement of the proximal fracture fragment may occur due to the triceps muscle pull in cases including a ruptured periosteum and triceps aponeurosis [3].

The standard treatment for displaced olecranon fractures is open reduction and internal fixation using tension band wiring or plate fixation [2, 5–7].

However, due to multiple medical comorbidities, low functional demands, increased surgical risks and medical complications like post-operative delirium in geriatric patients, displaced olecranon fractures have progressively been treated conservatively with reasonable results [1, 8, 9]. One prospective randomized study investigated the outcome between conservative and surgical treatment of displaced olecranon fractures in the elderly. This trial had to be stopped during the study period because of a high complication rate of 82% in the surgical group and further randomization was unethical [10].

This reported complication rate seems higher than obvious in everyday practice. Recently, a review article concluded that current data is not sufficient to evaluate the overall benefit of conservative treatment, yet it might serve as an option for selected patients in the elderly population [11].

Data seems scarce regarding the comparison between surgical and conservative treatment of displaced olecranon fractures in the geriatric population.

Therefore, the aim of this study was to evaluate the difference of the clinical outcome of elderly patients who were either treated conservatively or surgically for a displaced olecranon fracture (Mayo type IIA or IIB). The hypothesis was that there was no difference in the treatment outcome.

## Patients and methods

This retrospective follow-up study included patients above the age of 70 years who sustained a Mayo type IIA or IIB olecranon fracture between July 2015 and February 2019 and who were administered to the local community university hospital.

A radiograph search was conducted for all patients above the age of 70 years who sustained an elbow injury. All patients who showed undisplaced olecranon injuries (Mayo type IA and IB), dislocation fractures (Mayo type III A and IIIB) or any other elbow injuries were excluded. Only patients with a Mayo type IIA or IIB injury were included. The distance between the fractured fragments was defined to be at least 5 mm at the articular side to obtain a homogenous group of patients with a widely displaced olecranon fracture. All patients were invited to conduct clinical and radiological follow-up examinations.

Thirty-one patients were identified by radiographs who were eligible for inclusion. The patients were divided into two groups depending on their treatment method. Group I was treated conservatively by cast fixation or early functional treatment and group II was treated surgically. At the time of follow-up six patients were deceased, two patients suffered from end-stage dementia, four were not willing to participate because they were too frail and three were not reachable. Therefore, sixteen patients with seventeen olecranon fractures (group I *n* = 6 and group II *n* = 11; mean age 79 years, range 70–91 years; 12 female and 4 male) underwent a retrospective clinical and radiologic follow-up. The mean follow-up time was 23 months (range 12–45 months; group I 15 months (12–24 months)/group II 27 months (12–45 months)) after the injury. Formerly, a displaced Mayo type II fracture was usually treated surgically due to the displacement. With upcoming evidence that this type of fracture may be treated conservatively in geriatric patients, a paradigm change occurred in our department and geriatric patients were progressively treated conservatively. Therefore, most patients who were treated before 2018 were treated surgically and after 2018 rather conservatively. However, patient frailty, comorbidities and demands and requests were thoroughly evaluated in the cases after 2018 if surgical or conservative treatment would be best for the individual patient. Generally speaking, healthier and more active patients, who live independently, were rather treated surgically while frail and sick patients, who usually live in nursery home, were rather treated conservatively. Six fractures occurred in the dominant arm (group I *n* = 2; group II *n* = 4), nine in the non-dominant arm (group I *n* = 4; group II *n* = 6) and one patient was both-handed (group II). In group I treatment was inhomogeneous with either 5 weeks of upper arm splint immobilization, immediate functional therapy or short term upper arm splint immobilization (7–10 days) followed by functional therapy. The surgical treatment consisted of either plate fixation (*n* = 9) or tension bend wiring (*n* = 2).

The objective clinical measurement parameters were the active range of motion (ROM) of the elbow, the extension and flexion strength of the elbow and the grip strength for both sides. The range of motion was measured with the “Goniometer N400” (Biometrics Ltd, Newport, UK). Strength was assessed with the “Dynamometer G200” (Biometrics Ltd, Newport, UK). The patients were asked to squeeze the dynamometer and extend and flex the elbow against the dynamometer three times in a row for assessment. The mean out of the three measurements was used for calculations. The software “E Link SW2111-1196 Version 11.01” (Biometrics Ltd, Newport, UK) was used to assess these parameters.

The functional outcome was assessed using a german version of the “Disability of the Arm, Shoulder and Hand Score” (DASH), the “Oxford elbow score” (OES), the “Mayo Elbow Performance Index” (MEPI) and the Broberg and Morrey rating system. Pain was measured using the visual analog score (VAS) while resting and under load with 0 meaning no pain and 10 meaning the most severe pain.

Radiologic follow-up consisted of an anteroposterior and lateral radiograph of the elbow. The distance between the proximal and distal fracture fragment at the articular side and the posterior cortex were measured using the Impax EE R20 viewer (Agfa Healthcare, Mortsel, Belgium) on initial and follow-up radiographs.

Any complications during the healing process and comorbidities were recorded from the patient`s chart data.

The data was de-identified primarily. Data was recorded and analyzed using Microsoft Excel (Version 2016, Microsoft Corporation, Redmond, Washington, USA). Because of the low patient count, non-parametric tests (Mann–Whitney *U* Test and Fisher´s Exact Test) were used for comparative calculations using SPSS (Version 24.0, IBM, Armonk, NY, USA). Statistical significance was set at *p* < 0.05. Data are presented using descriptive statistics.

## Results

**Fig. 1:**
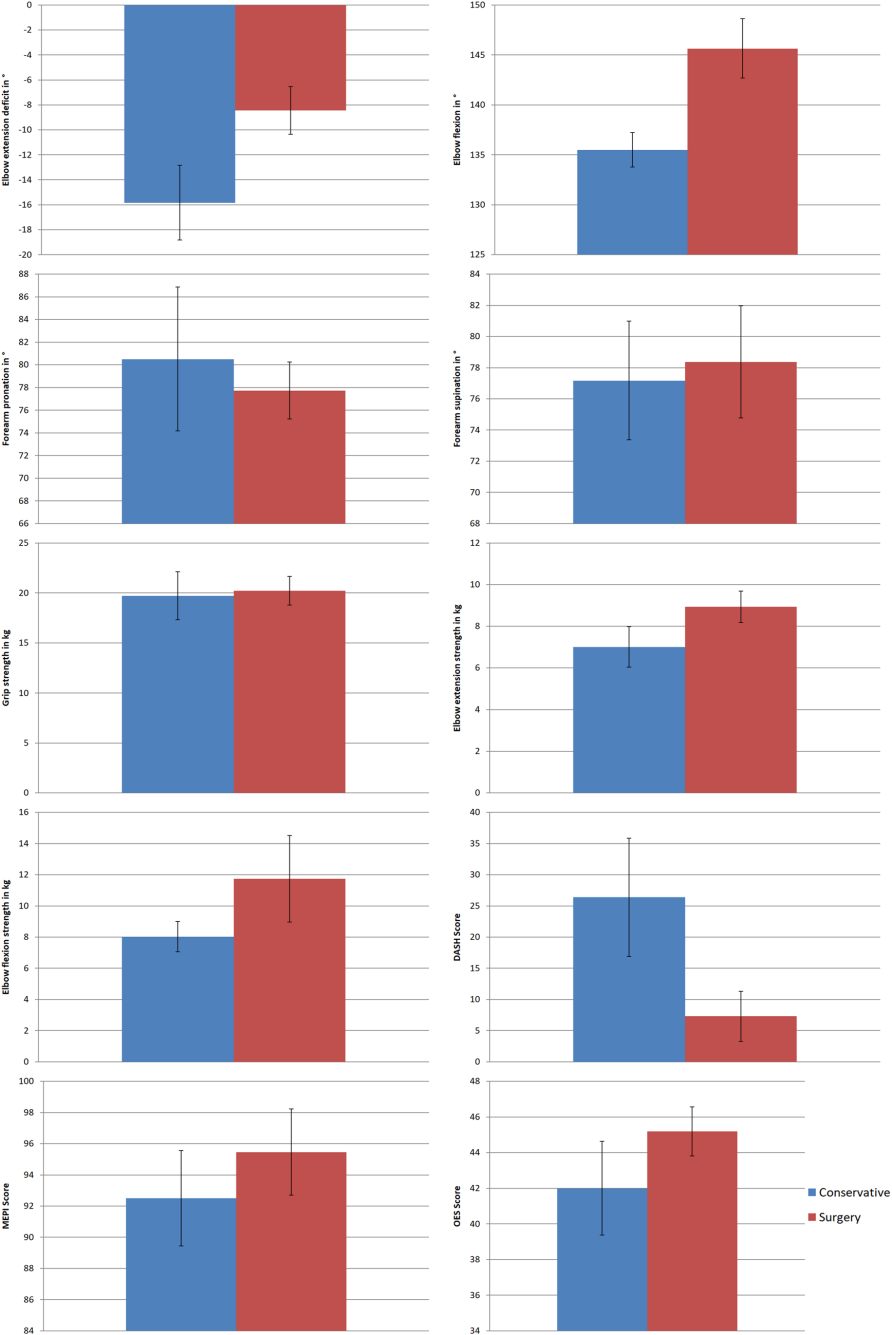
Illustration of the difference of the results for the range of motion, strength measurements and functional scores for the conservative and surgical group (graphs show the mean and standard error)

**Fig. 2:**
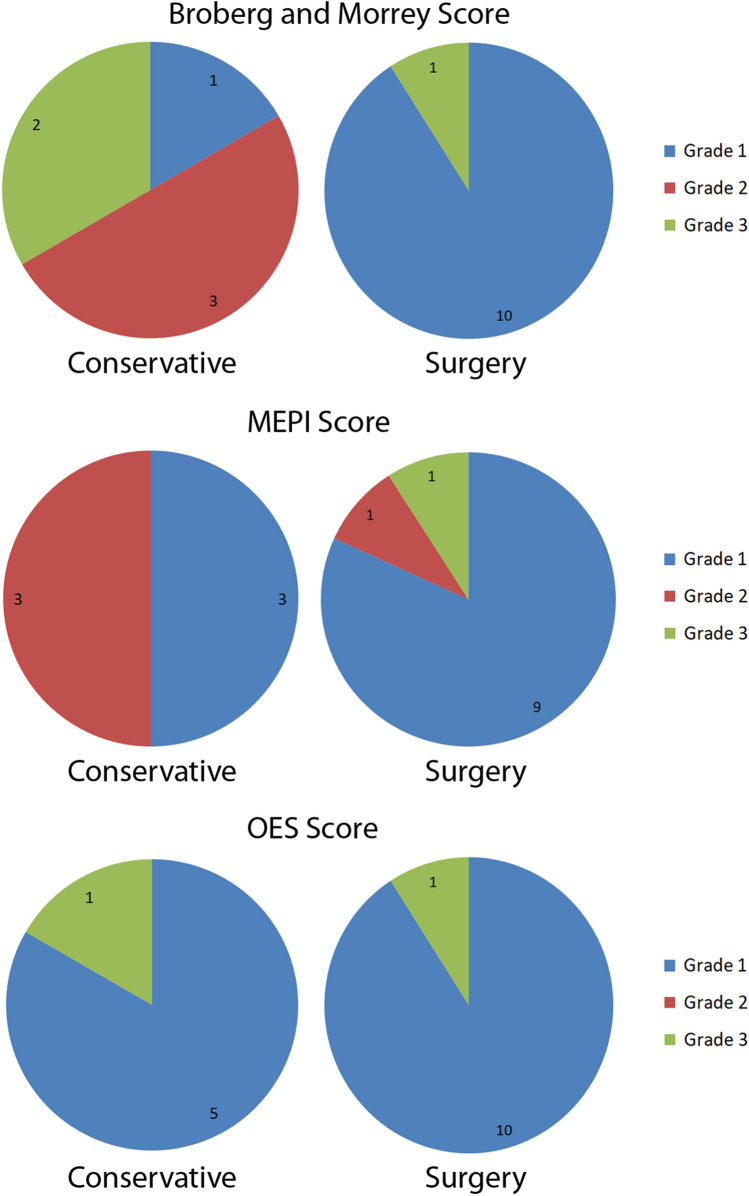
Grading results of the functional scores for the conservative and surgical group

The range of motion, the arm strength, the pain level, the functional scores, the size of the initial fracture gap distance and the mean age at injury are shown in Table 1, Figs. 1 and 2. The arch of motion was 120° in the conservative group and 136° in the surgical group. There was no obvious difference in elbow extension strength in comparison to the healthy contralateral side (*p* = 0.20; 88% group I/87% group II). There was no difference in the OES (*p* = 0.30; 42 (SD 7) vs. 45 (SD 5)) and MEPI score (*p* = 0.46; 93 (SD 8) vs. 96 (SD 19)) between both groups. The conservative group presented a slightly worse DASH (*p* = 0.10; 26 (SD 25) vs 7 (SD 14)) and a significantly worse Broberg and Morrey score (*p* = 0.02; 84 (SD 9) vs. 95 (SD 7)) than the surgical group. Conservatively treated patients were significantly older than surgically treated patients (*p* = 0.007; 85 (SD 4) vs. 76 (SD 6) years).

**Table 1:** Outcome results for the conservative and surgical treatment groups

Measurement parameter	Conservative treatment (Mean (SD)/% compared to contralateral side); *n* = 6	Surgical treatment (Mean (SD)/% compared to contralateral side); *n* = 11	*p* value
Elbowextensiondeficit	− 16° (8°)/214%	− 9° (SD 7°)/490%	0.048
Elbowflexion	136° (5°)/99%	145° (10°)/100%	0.06
Forearmpronation	81° (17°)/96%	78° (9°)/105%	0.26
Forearmsupination	77° (10°)/102%	78° (13°)/93%	0.40
Gripstrength	19.7 kg (6.0 kg)/89%	20.2 kg (4.9 kg)/94%	0.96
Elbowextensionstrength	7.0 kg (2.4 kg)/88%	8.9 kg (2.5 kg)/87%	0.21
Elbowflexionstrength	8.0 kg (2.4 kg)/100%	11.7 kg (9.2 kg)/87%	0.44
BrobergandMorrey score	84 (9)	95 (7)	0.20
MEPI score	93 (8)	96 (10)	0.46
OES score	42 (7)	45 (5)	0.30
DASH score	26 (25)	7 (14)	0.10
VAS (Rest)	0 (0)	0 (0)	1.00
VAS (Load)	1.0 (1.3)	0.5 (1.2)	0.35
Initial fracture gap (dorsal rim)	22 mm (10 mm)	18 mm (6 mm)	0.53
Initial fracture gap (ventral rim)	10 mm (4 mm)	9 mm (4 mm)	0.46
Meanage at injury	85 years (range 80–91 years)	75 years (70–90 years)	0.007

All patients were very satisfied in both groups except one patient in the conservative group who was only partially satisfied. This patient suffered from an ulnar nerve palsy with dysesthesia. This was the only complication in the conservative group (9%). The surgical group presented two complications (18%). One patient suffered from a prolonged lymphedema because of a previous breast cancer surgery. A second patient needed a revision surgery because of a long screw that blocked forearm rotation. Implant removal was conducted in four other patients (36%). All patients suffered from multiple different comorbidities except one in the surgical group who had no comorbidities except a St.p. thyroidectomy.

After fracture healing there was no gap or non-union in the surgical group. The conservative group showed a non-union with a persistent fracture gap of 13 mm (SD 8 mm) at the articular rim and 27 mm (14 mm) at the dorsal rim.

**Fig. 3:**
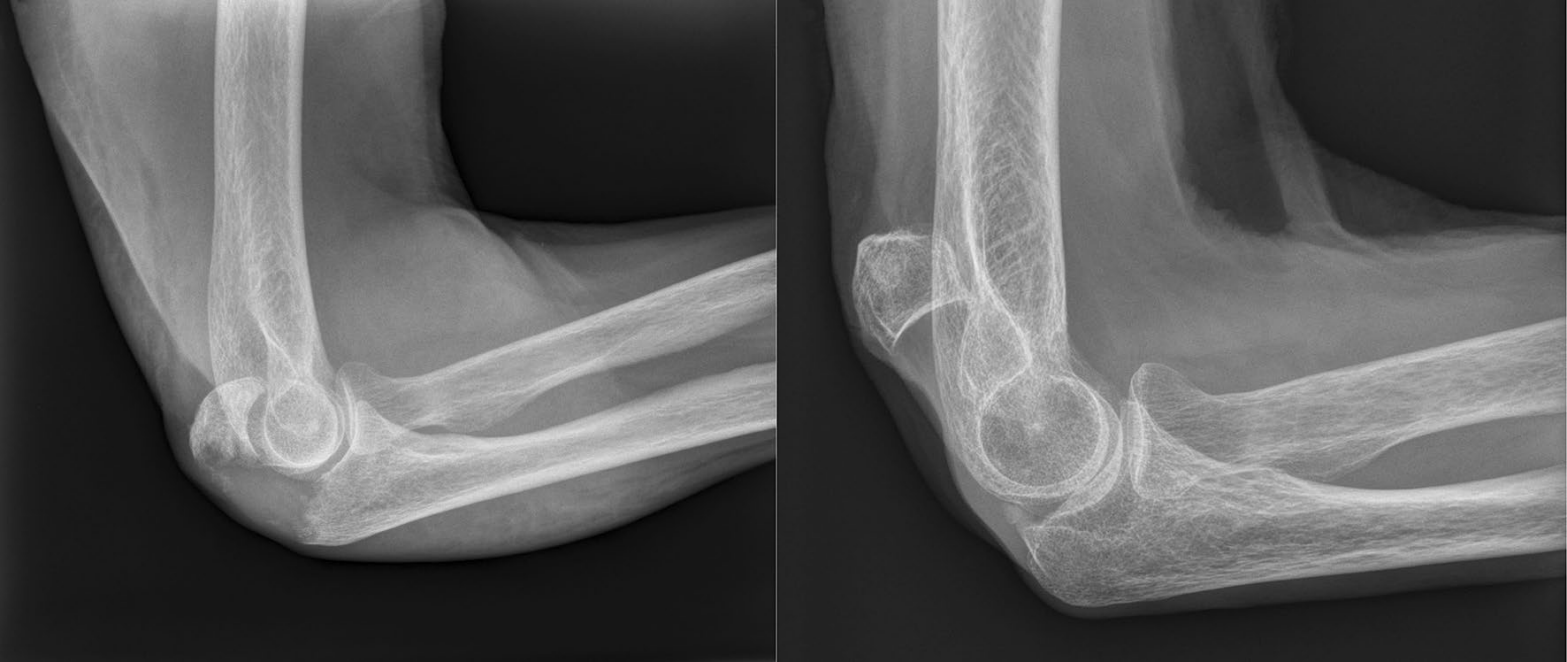
Lateral radiographs of a patient´s olecranon fracture (Mayo type IIA) on the injury day and at follow-up

**Fig. 4:**
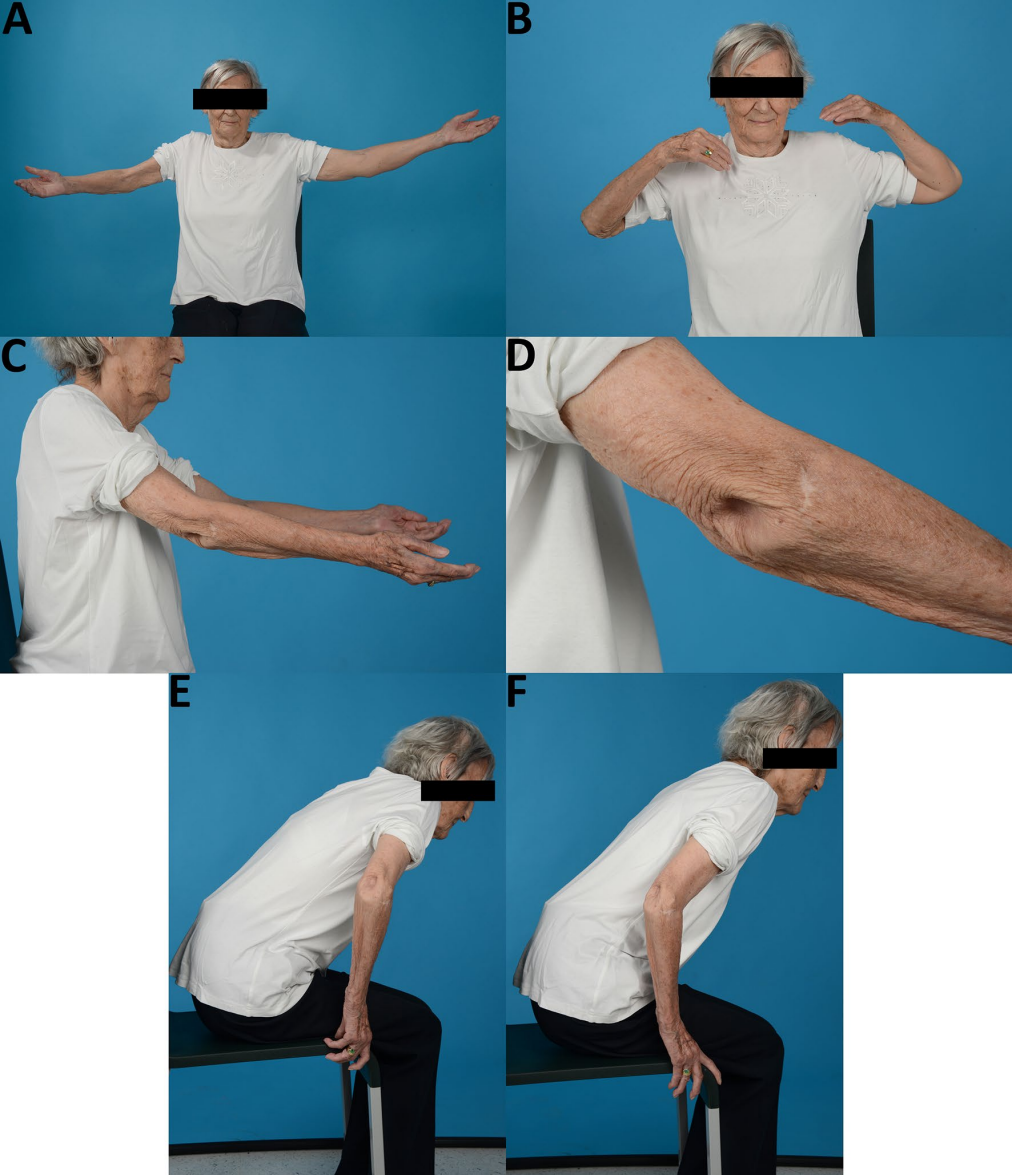
The same patient of Fig. 3 presenting elbow extension (**A**), elbow flexion (**B**), side view of elbow extension (**C**), dorsal olecranon region (**D**), the use of elbow extension in rising up from a chair (**E** + **F**)

An exemplary case of a patient who sustained a displaced olecranon fracture is presented in Figs. 3 and 4.

## Discussion

The most important finding of the present study was that both treatment methods (surgical and conservative treatment) for widely displaced olecranon fractures led to satisfied elderly patients with a good elbow function.

Recommendations for the treatment of olecranon fractures remain controversial. On the one hand, surgical treatment of displaced olecranon fractures is widely conducted [2, 3, 12, 13], although the surgical risk can be as high as 70–82%, especially in osteoporotic bone and difficult soft tissue conditions [10, 14]. On the other hand, retrospective studies showed that Mayo type II fractures heal with reasonable results following conservative treatment in the low-demanding geriatric patient population without the necessity to treat non-unions surgically [9, 15–17]. Additionally, a recent prospective randomized study showed that there was no statistical significant difference in the DASH, MES, VAS and Broberg and Morrey score between surgically and conservatively treated patients [10]. Our results for conservatively treated patients were similar regarding these scores except for a higher DASH score for the surgical group in our study. Additionally, the authors saw a significantly diminished mean arc of motion in the conservative group similar to our results (120° vs. 136°). Conservatively treated patients showed an approximately 15° increased extension deficit compared to surgically treated patients. This may have an influence on the functional scores that were all reasonable, yet slightly worse in the conservative group when regarding the grading (Fig. 2). The higher extension deficit in the conservative group may be explained because of intraarticular remodeling, limiting scar tissue and skin adhesions (Fig. 4D).

However, a loss of elbow extension of more than 10° was also present in up to 40% of all surgically treated patients with a displaced olecranon fracture in a retrospective analysis [18]. Most activities of daily living can be accomplished with an active elbow flexion arc of 100° (0°–30°–130°) and 100° of forearm rotation (50°–0°–50°) [19]. Therefore, some minor loss of elbow extension does not seem to significantly influence the patients` satisfaction and functional outcome negatively.

A recent systematic review of four included studies described similar findings to the present study with a mean MEPI socre of 95, a mean DASH score of 12, a mean flexion of 133° (106–140°), a mean extension lag of 15° (0–30°) and a mean VAS score of 1 (0–8) [20]. The authors conclude that the available literature supports a consideration of nonoperative treatment in the low-demanding elderly patients [20].

Interestingly, the elbow extension strength did not differ compared to the contralateral side in both groups. The reason may be because the overall extension strength in the low-demanding population is low in general. The extension force may be produced and compensated by other pericubital extending muscles and additionally transmitted via the brachial and antebrachial fascia. Yet, the results can only be applied to the low-demanding geriatric population; results ma potentially vary significantly when investigating young and athletic individuals.

Pain did not seem to be an issue in both groups similar to Duckworth et al. [10]. Even in highly displaced olecranon non-unions there was also no joint degeneration leading to significant pain. However, one patient who was only partially satisfied suffered from ulnar nerve neuropraxia. The displaced olecranon fragment may have potentially irritated the ulnar nerve and lead to this ulnar nerve syndrome, yet this patient did not want to have further treatment. In this context, it is worth to mention that the patients who were not willing to participate in the study or for whom relatives reported on their dementia or death, the elbow did not seem to play a significant problem in everyday life, which needed evaluation or treatment in the eyes of the patient or their relatives. As seen in our patient example (Figs. 3 and 4), a reasonable elbow function can be achieved despite a widely displaced olecranon fragment.

Although conservatively treated patients showed a good clinical outcome, the functional scores seemed lower than in the surgically treated patients. One reason might be that both cohorts potentially differed regarding their age and potential overall fitness. The surgical group was slightly younger, therefore potentially healthier, and assumably less frail than the other group. A similar finding was present in Duckworth´s study with a slightly older population in the conservative group of patients [10]. The fracture morphology and the displacement were not different and therefore did probably not play a role in our study. Another reason for this finding may certainly be the treatment method itself that may lead to a better function and therefore a better score. Yet, the high MEPI reflected the patient’s good elbow function. A closer look on the DASH score revealed that the reported impairment was due to the patient’s overall frailty, as activities of daily living could be performed with none or only minor difficulties. Other previous unknown injuries or disabilities of the shoulder for example may also have an influence on these functional results.

Surgery can lead to complications and reoperations. A prospective randomized study was stopped because the complication seemed too high in the surgical group [10]. The reported reasons were a loss of reduction in six patients and one superficial wound infection. Our study did not see such a high complication rate. The reason may be due to a different surgical technique. While almost all patients were treated with plate fixation in this study, the aforementioned paper used mainly tension band wiring as the treatment method. Although not investigated, one can assume that because of the usually poor bone quality in elderly and geriatric patients, tension bend wiring is not a good surgical option because this implant has no firm hold in the bone. This technique may therefore lead to loss of reduction. Locking plates may decrease this complication as they lead to a tighter fixation and can be applied as an additional buttress against post-operative fracture fragment displacement. Another reason for surgical failure and a higher failure rate in other studies may be the size of the fragment. Small avulsion fragments are difficult to address and retain than bigger ones. Bigger fragments usually have better screw and k-wire purchase. In this study, a homogenous group of patients with a big fracture fragment including more or less half of the articular surface similar to Fig. 3 were included.

Another issue in surgery is implant removal. It has been described to be necessary in up to 65%-80% of all surgically treated cases in geriatric patients because of skin irritation, wound breakdown or pain [21]. In this study, implant removal was necessary in four patients (36%) because of local irritation and discomfort. Yet, all patients were satisfied thereafter and did not show any surgical or anesthetic complications.

However, the geriatric population typically suffers from multiple comorbidities, a high American Society of Anesthesiologist grade (ASA), frailty and poor bone quality [12, 15], which might influence anesthesia, surgery and medical after care and may naturally lead to more complications, a higher morbidity and mortality [22–25].

Frailty itself includes low activity and low demands in geriatric patients, which might be a reason for a lower functional demand of the elbow joint and therefore the high satisfaction rate in this study irrespective of the treatment choice. Therefore, it seems essential to carefully balance surgical treatment risks and potential benefits against conservative treatment risks and benefits, especially with regard to the patient’s demands and requests.

The results of this study need to be regarded critically. The patient number is low in both groups, however, it seems difficult to acquire more patients as they are either too frail to participate or decease few months to years after the injury. This is the main limitation of the presented study. However, one can argue that because of this fact, conservative treatment may be beneficial in this cohort, as the functional results seem good, the patients are satisfied and the complication risks are low, irrespective of a potential slightly better functional outcome after surgery. Another limitation is the fact that the surgical group was significantly little bit younger than the other group. Potentially the conservative group was frailer and the younger group more active. Therefore, it cannot be answered if the surgical group would also be similarly satisfied or show similar good results if treated conservatively. Similar to other studies [20], there might have been a high risk of selection bias regarding the choice of treatment in our study, as younger patients were rather treated surgically and older ones conservatively. Up to date, no relevant conclusions can be drawn when comparing conservative treatment with surgical treatment, especially in regard to more active elderly patients and additional research with randomized controlled multicenter trials involving larger sample sizes and longer follow-ups are necessary [20].

## Conclusion

This study showed that widely displaced olecranon fractures can successfully be treated conservatively in low-demanding geriatric patients with a god and satisfactory outcome. However, patient selection is essential because patients that are more active might benefit from surgical treatment. Yet, treatment risks and benefits need to be balanced carefully in regard to patient`s demands and requests.
